# Patient and Family Engagement Approaches for Digital Health Initiatives: Protocol for a Case Study

**DOI:** 10.2196/24274

**Published:** 2021-07-21

**Authors:** Nelson Shen, Damian Jankowicz, Gillian Strudwick

**Affiliations:** 1 Centre for Addiction and Mental Health Toronto, ON Canada; 2 Institute of Health Policy, Management and Evaluation University of Toronto Toronto, ON Canada

**Keywords:** digital health, patient engagement, case study, patient and family engagement, mental health

## Abstract

**Background:**

Digital health initiatives such as patient portals, virtual care platforms, and smartphone-based apps are being implemented at a rapid pace in health care organizations worldwide. This is often done to improve access beyond traditional in-person care and enhance care quality. Recent studies have indicated that better outcomes of using these initiatives and technologies may be achieved when patients and their family members are engaged in all aspects of planning, implementation, use, and evaluation. However, little guidance exists for how health care administrators can achieve effective engagement in digital health initiatives specifically.

**Objective:**

The objective of this study is to document processes related to planning and implementing patient and family engagement (PFE) in digital health initiatives. This information will be used to develop tangible resources (eg, a field guide) that other organizations can use to implement PFE approaches for digital health initiatives in their organizations.

**Methods:**

A previously developed multidimensional conceptual framework for PFE in health and health care contexts will be used to guide this work. To understand the intricacies involved in using PFE approaches in digital health strategies, a case study will be conducted. More specifically, this work will employ an embedded single-case design with PFE in digital health initiatives at a large Canadian mental health and addictions teaching hospital. Multiple digital health projects being undertaken at the study site will be explored to better understand where the PFE is intended to support the design, implementation, and operation of the digital health platform or technology. These projects will form the individual units of analysis. Data collection will involve field notes and artifact collection by a participant observer and interviews with the various digital health project teams. Data analysis will include a content and thematic analysis, triangulation of the findings, and a chronological mapping of data to a PFE process.

**Results:**

Funding for this work was provided by the Canadian Institutes of Health Research (CIHR), via a Health System Impact Fellowship. As of August 2020, digital health projects that will form the case study units have been identified, and the participant observer has started to embed themselves into these projects. Although the development and collection of field notes and artifacts, respectively, have begun, interviews have not been conducted. The study is expected to conclude in September 2021. Once this study is complete, the development of a field guide and resources to support the uptake of PFE strategies in digital health will begin.

**Conclusions:**

By better understanding the processes involved in PFE in digital health projects, guidance can be provided to relevant stakeholders and organizations about how to do this work in an effective manner. It is then anticipated that with the increasing use of PFE approaches, there may be improved uptake, experience, and outcomes associated with using digital health technologies.

**International Registered Report Identifier (IRRID):**

PRR1-10.2196/24274

## Introduction

### Background

The potential for digital health initiatives, such as electronic health record systems, mobile health apps, and virtual care platforms, to have a positive impact on aspects of health care delivery, including in effectiveness and patient safety, is well documented in the literature [1-4]. In this paper, digital health initiatives refer to several digital technologies including, but not limited to, mobile apps, online digital interventions, telemedicine or virtual care platforms, electronic health records, and patient portals [1]. These technologies may empower patients, providers, and other knowledge users by providing them with information to improve decision making in self-management, point of care, health policy, and beyond. In addition, some of these technologies (eg, virtual care platforms) may allow for improved access to care beyond the physical location in which it is typically delivered.

Numerous health care organizations have already implemented digital health initiatives; however, there have been varying degrees of success. A recent systematic review found that 81% of evaluations of these technologies showed significant improvements in efficiencies or effectiveness due to the adoption of the technology, with the remaining studies showing mixed or negative results [5]. Despite the potential benefit, many digital health initiatives have faced implementation barriers, hindering their update and adoption (eg, technology issues, mismatched values, changes in workflow, organizational culture issues, etc) [6]. Lessons learned from failed initiatives suggest that the active involvement of patients in the various aspects of developing and implementing digital health initiatives (or patient engagement) is critical to its success, especially those targeted for use specifically by patients and family members themselves [7-9]. Studies have shown positive outcomes when patients are engaged throughout the lifecycle of health technology projects [10-12]. Recently published work describing digital health initiatives showed that engaging patients in the design and implementation process has yielded positive results [12,13].

A 2019 scoping review exploring patient and family engagement (PFE) strategies in health information technology (IT) initiatives found that engaging patients and families has become a growing trend in recent years, with most (57%) of the included studies in that review being published since 2017. The review also found that there are varying degrees of engagement of patients and family members by health care organizations, with often limited engagement sustained throughout the entirety of the stages of digital health projects [14]. A recent Canadian study was conducted to understand how health care organizations can develop strategies to facilitate greater patient engagement in their health IT initiatives [15]. A core recommendation of the study was to develop a resource document, or guide, to support health care organizations in developing and integrating patient engagement strategies in digital health initiatives.

Although the increasing trend of engaging patients and families in digital health initiatives remains apparent, there lacks a validated patient engagement field guide or resource to inform administrators of effective strategies. A 2018 scoping review on patient engagement in Canada called for greater investments directed to the development, validation, and implementation of a practical patient engagement resource [16]. Thus, there is consensus on the importance of patient engagement beyond direct care (ie, shared decision making) to include patient partnerships in decision making at the organizational and policy level. For this reason, a validated patient engagement field guide is necessary to promote patient decision making at the organizational and policy level and to assist digital health initiatives in sustaining patient engagement. To develop this kind of resource or guide, more information is required to understand the key processes involved in engaging patients and families in digital health initiatives. The purpose of this study is to use a case study approach to generate an understanding of how PFE strategies are initiated and integrated into digital health initiatives at a specialty Canadian academic health science center.

### Conceptual Frameworks

The concept of patient engagement is being increasingly accepted in health care; however, it takes on different meanings for different stakeholders in different settings [17,18]. To provide some conceptual clarity, Carmen et al [19] proposed a multidimensional framework for PFE in health and health care. This framework has been used in health IT projects to inform PFE strategies in health IT adoption, implementation, use, selection, and evaluation [14]. The framework identifies a continuum of engagement that ranges from consultation, to partnership, to shared leadership. These 3 categories of engagement can occur at 3 different levels of the health care system: direct care, health care organizational design and governance, and policy making [19]. The current study will be framed in the context of PFE in organizational design and governance, and policy making, representing PFE in the design of the digital health technologies, processes, and policies at an organizational or systems level. Although PFE in direct care through digital health can empower and equip patients for better self-care, opportunities exist to explore and understand the meso- or macrolevel PFE contexts [20].

**Figure 1:**
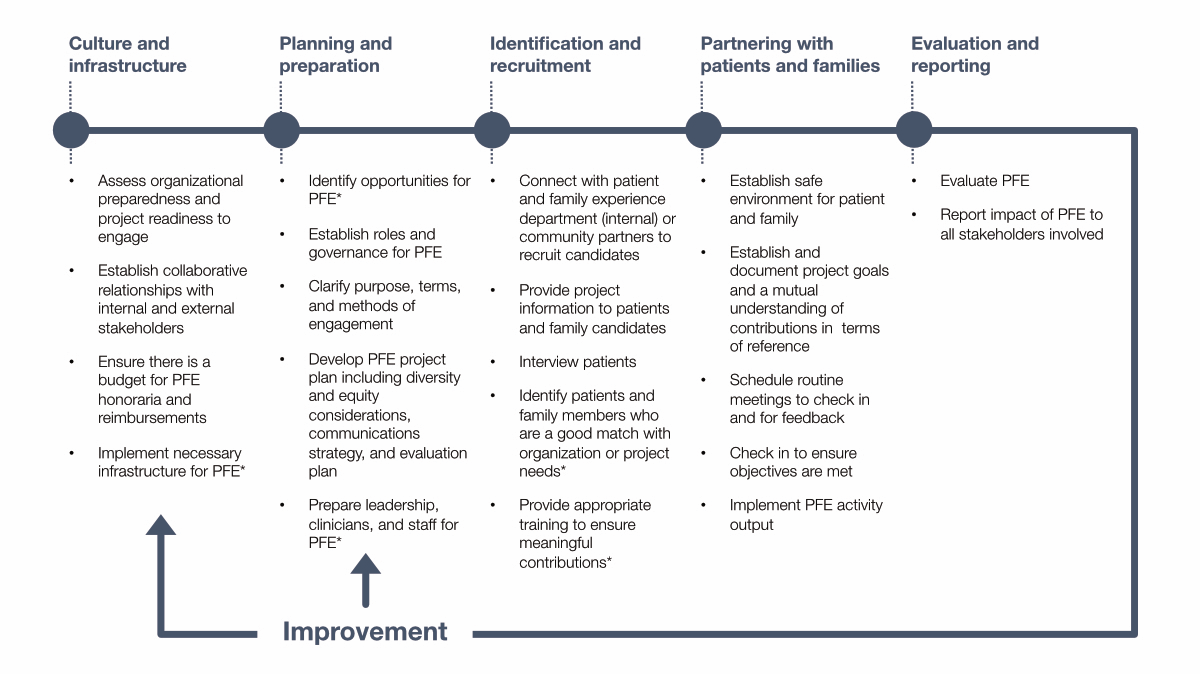
Patient and family engagement process map: PFE: patient and family engagement. * denotes steps identified by the Agency for Healthcare Research and Quality guide [22].

Although the Carmen framework provides types of PFE nested in different health care contexts, it does not offer guidance on the process of implementing PFE initiatives. Existing efforts to provide this guidance have come in the form of lessons learned, recommendations, and checklists; however, a more robust understanding of how to engage patients efficiently, effectively, and meaningfully is required [21]. A brief scan of academic and gray literature identified only a few resources that provide an in-depth guide to PFE [22-26]. The implementation process by the Agency for Healthcare Research and Quality [22] was selected to be the foundation, as it outlines practical in-depth guidance on implementing PFE to support hospital quality improvement and safety initiatives. To provide a more comprehensive process map, some adaptations were made to the process, such as establishing a culture for PFE, evaluation, and the cyclical nature of the process [23-26]. This preliminary process map was further adapted with a systematic scan of PFE tool kits identified in the gray literature. This iteration of the PFE process map (Figure 1) includes the information from the Canadian tool kits and guides identified [23,25,27-34]. The next iterations of the map will be validated through consultation with the PFE teams at the study site and will be used as a framework for this case study.

## Methods

### Overview

This study is part of a larger project aimed at developing a field guide to support effective patient and family engagement in digital health initiatives. This project is funded through the Canadian Institute of Health Research Health (CIHR) System Impact Fellowship, where the objective is for embedded fellows to use an integrated knowledge translation approach to advance an organization’s impact goals regarding health system improvement [35]*.* This study will take place at a mental health and addictions–focused Canadian academic health science center, where PFE was identified as a guiding principle in the 3-year strategic plan for the center and its digital innovation strategy. As such, the focal point of this project is to build capacity for PFE at the center. This project was approved as a quality improvement project by the Quality Project Ethics Board at the center and will be conducted between December 2020 and September 2021.

A case study will be conducted to understand the intricacies involved in employing patient engagement activities in digital health initiatives. Case studies typically involve the analysis of an object of study within a real-life, contemporary context or setting [36]. It is an exploration of a bounded system (a case) through detailed, in-depth data collection involving multiple sources of information (ie, observations, interviews, and artifacts) [37,38]. The resultant report will describe the experience and discuss themes within the experience.

This case study asks the following: what are the necessary elements to support effective PFE engagement in digital health initiatives? To answer this question, this project has 7 objectives: (1) map current PFE processes, (2) identify key steps for PFE, (3) understand digital health team needs in planning and implementing PFE, (4) understand the staff experience, (5) understand patient experience, (6) identify ways to improve PFE planning and implementation, and (7) identify core artifacts that support PFE organization and implementation.

**Figure 2:**
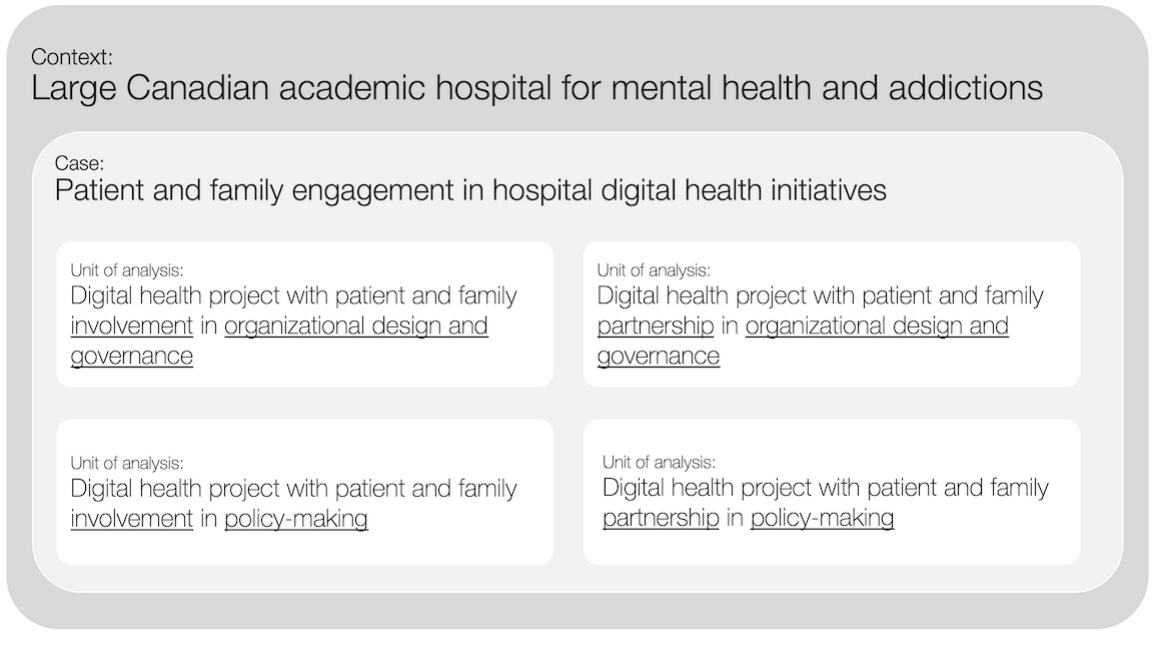
Embedded case study design.

This project will employ an embedded single-case design with PFE in digital health initiatives at the mental health and addictions academic health science center (see Figure 2). Within this case, this study will explore the development and implementation of PFE in multiple digital health projects, where the PFE is intended to support the design, implementation, and operation of the digital health platform or technology. Each project engaged in this case study will be a unit of analysis. Within each unit of analysis, a member of the research team will be embedded as a participant observer to support the project team in organizing and facilitating PFE activities while documenting the processes and tacit knowledge required through field notes and artifact collection. Interviews with each digital health team will be conducted to understand the experience and to triangulate with the observations.

The motivation for this case study approach is instrumental and takes on a praxeological lens or action-based approach in understanding how PFE processes are conducted and how they can be improved [39]. The findings from this case study are intended to integrate first-hand experiential knowledge into the development of the field guide. As primary users of the field guide, this case study aims to understand the digital health project team’s information needs and lessons learned through the projects.

### Recruitment

A maximum variation purposive sampling strategy will be employed to identify digital projects at the academic health center. This strategy will be employed to identify cross cutting themes derived through a diverse range of experiences in implementing PFE. Recruitment will be based on the Carman et al [19] framework and will maximize variation across the different organizational domains (ie, “organization design and governance” and “policy making”) and levels of engagement (ie, “involvement or “partnership”). This protocol acknowledges that recruitment will be dependent upon the types of projects available and that PFE activities at the higher levels of the engagement spectrum, as proposed here, are less frequent [40,41]. Depending on these factors, a convenience sample may be required.

Digital health projects will be identified through consultation with departmental leadership and the Digital Health Steering Committee at the academic health center. Project leads identified through consultation will be contacted via email to schedule a meeting to discuss their project and to assess the fit of the project for this case study. Preference will be given to projects that are still in their early planning stages of integrating PFE, as it would allow the research team to observe and participate in the entire process of organizing and implementing PFE in a project.

### Data Collection

A key characteristic of a case study is the collection and convergence of multiple data sources to allow for a holistic understanding of the case [42]. This case study will employ participant observations and interviews as the 2 primary types of data collection methods which will yield 4 types of data: field notes, project artifacts, documentation, and interview transcripts. Table 1 shows the linkages between the data sources and research questions. In light of the COVID-19 pandemic, all data collection will be facilitated electronically through institution-approved email, cloud drives, and videoconferencing platforms.

**Table 1:** Linkages between case study objectives and data types.

Case study objectives	Data types			
	Field notes	Artifacts	Documents	Interview transcripts
Map current PFE^a^ processes		✓	✓	✓
Identify key steps for PFE	✓			✓
Understand digital health team needs in planning and implementing PFE	✓			✓
Understand the staff experience	✓		✓	✓
Understand patient experience			✓	✓
Identify ways to improve PFE planning and implementation	✓	✓	✓	✓
Identify core artifacts that support PFE organization and implementation	✓	✓		✓

^a^PFE: patient and family engagement.

#### Participant Observations

A member of the research team (NS) will be embedded as a participant observer in the projects. As a participant, NS may assume a variety of roles on the digital health project and be actively involved in the planning and implementation of the PFE activity. As an observer, NS will take field notes following an observation protocol (see Multimedia Appendix 1 for these protocols). The field notes will document conversations related to the PFE activity and the processes involved in planning and implementing PFE activity (eg, questions asked about PFE and process, decision points, information requirements). Notes related to implementation factors will also be documented as they are critical to understanding the processes involved (ie, enablers, barriers, pain points, and breakthrough points). Additionally, physical and digital artifacts used throughout the projects will be collected. These artifacts are items that are used to facilitate planning and implementation (eg, technologies, templates, patient-facing material, process maps, project documents, PowerPoint slides decks). Documentation related to the project may also be used to provide context to the cases. Documentation includes agendas, meeting minutes, report of events, proposals, progress reports, evaluation reports, published articles, and news appearing in institutional announcements and mass media. [38].

#### Interviews

Semistructured interviews will be conducted to provide greater insights and context to the PFE processes and informational needs of digital health teams. Approximately 12 interviews will be conducted across the 4 digital health projects and will follow an interview script (see Multimedia Appendix 1). Interviews will be conducted with the project lead or manager and the staff supporting the project. Additionally, patient and family advisors will also be interviewed in projects where they are a member of the digital health team (ie, participating in meetings and contributing to the digital health project). Staff will also be asked about their current knowledge and knowledge gaps regarding the planning of PFE activities. The interviewer will ask participants about their experiences planning and implementing their PFE activities, what was successful, what they would do differently, and lessons they learned throughout the process.

The interviews will be conducted with the teams at the conclusion of a PFE activity or event. For projects with ongoing PFE, these interviews will be conducted after a project milestone has been met or at other logical end points. All interviews will be conducted and audio recorded via a web-based video or audio platform approved by the organization. Field notes will also be taken.

### Data Management and Analysis

The data from the participant observations and interviews will be analyzed following a common data analysis method consisting of sequential deductive and inductive approaches. Data will first be deductively coded using a codebook derived from the PFE process map, where each step will be used as a code. Data that do not fit the scheme will be inductively coded as new categories.

The data collected in the participant observations will be analyzed using a qualitative descriptive approach with content analysis, which will provide a comprehensive summary that provides factual description of the data [43,44]. Data will be analyzed using a directed content analysis [45] that follows the sequential deductive-inductive approach. Relevant sections of the field notes will be coded line by line. Artifacts will be categorized based on where they were used in the process map.

The interview data will be transcribed verbatim and will be thematically analyzed following Braun and Clarke’s framework [46]. The framework consists of 7 steps: transcription, reading and familiarization, coding, searching for themes, reviewing themes, defining and naming themes, and finalizing the analysis. The coding process will also follow the deductive-inductive approach. Using NVivo 11 (QSR International), the analysis will be conducted in pairs, where the independent coding will be compared and discrepancies will be resolved through discussion.

A case description strategy will be used to organize the participant observation and interview results into a descriptive framework [38]. The results will be triangulated to cross-validate the findings and provide a comprehensive understanding of PFE [47]. This will be accomplished through a journey mapping technique that will visually plot the results on a timeline (ie, a PFE process map will be used as the template) [48]. The data collected through participant observation will undergo a time series analysis in which a chronological sequencing technique will be used to trace the data over the timeline for each project [38,49]. Critical milestones and decisions points within the process will be identified through commonalities in the field notes across multiple projects. Artifacts and key documents will also be mapped to the process based on when they would be relevant. Similarly, insights from the interviews will also be mapped to the journey map where they would be relevant. The comparison of multiple units strengthens the inferences that can be made about the process while addressing threats to internal validity; furthermore, the final journey map will undergo a round of member checking by the project teams and validation by the PFE teams. This final step will be used to identify any oversights and resolve any contradictions [38].

## Results

Three digital health projects that will form the case study units have been identified, and the participant observer has started to embed themselves into these projects. One project used a consultation approach to PFE; the other two projects used multiple approaches. One involved consultation and involvement, while the other had all three approaches of consultation, involvement, and partnership. The interviews have not been conducted and are anticipated to occur in Spring 2021. This study is expected to conclude in September 2021. Once this study is complete, the development of a field guide and resources to support the uptake of PFE strategies in digital health will begin.

## Discussion

### Principal Findings

Digital health is increasingly gaining prominence in today’s health care landscape. Its importance has been emphasized with the COVID-19 pandemic, during which health care organizations and providers are leveraging virtual and digital means to coordinate a response to the unprecedented challenge [50]. Engaging patients ensures the valid design of patient-centered digital health innovations, where the discrepancy between user and clinical realities are minimized [51]. Furthermore, patient engagement in the ideation, development, implementation, and governance in new digital health innovations is critical in addressing the social, cultural, technological, and ethical challenges of digital health implementation [52]; moreover, it ensures that the end product and the processes that support it are desirable, feasible, and viable for all stakeholders involved [53]. This case study aims to identify all the critical factors to ensure that PFE can be implemented effectively and meaningfully to support digital health innovations.

The findings from this proposed case study can fill an identified knowledge gap in health informatics and PFE literature [14,21]. This case study builds upon the work of a scoping review [14] and a stakeholder engagement activity [15] on how to facilitate PFE in digital health initiatives, cross-validating the recommendations from these previous works through the practical experience of implementing PFE strategies. As identified in the work to date, there is currently no common framework or resource to support health care organizations through the implementation of PFE strategies in their digital health initiatives. Taking a case study approach allows for the convergence of multiples sources of data and perspectives in developing a framework.

This protocol was published for the following reasons. First, the peer-review process offers the opportunity to improve the quality and applicability of this work through the insights of the reviewers. Second, this paper will inform the scientific community that this research is underway and to encourage collaboration from those interested. Last, publishing study protocols has the benefit of disseminating contemporary ideas and approaches to the complex problems [54]. Although case study research has been increasingly used by researchers, publishing a case study is important, as it is a relatively underused form of inquiry that is valuable for untangling dynamic and complex topics [55,56]. This protocol is intended to highlight how a case study approach can be pragmatically used to map out processes and form a foundation for developing a field guide.

Through the work outlined in this protocol, this case study will adapt existing processes for planning and implementing PFE for the digital health context. Future work will engage digital health teams, patients, and families in the co-design of the field guide. The field guide addresses an identified knowledge gap and will encourage greater PFE in digital health initiatives, thereby also addressing a practice gap in a field where adoption, scale, and spread are challenging.

### Limitations

This study has a few anticipated limitations to consider. First, the case study will be undertaken in a very specific context, where the digital health initiatives are focused on mental health and addictions and are implemented in an academic hospital setting. Moreover, the findings from this study will not be generalizable to a broader setting; however, this study is intended to be exploratory, providing a preliminary understanding of the process nuances and key steps rather than seeking causal relationships [57,58]. Future work will include cross-validating the findings and recommendations through an environmental scan of past digital health PFE projects across Canada (and perhaps beyond), offering the opportunity to extend the understanding of PFE processes in digital health. Second, the proposed PFE process presented in this protocol is still in a preliminary state and will undergo further adaptations based on other PFE guides identified throughout the case study. The adapted PFE process map will be further validated by the PFE teams to ensure a comprehensive and valid PFE process is available for the proposed time series analysis. Moreover, the PFE teams will be engaged throughout the case study to provide external validation of the interview and participant-observer findings. Finally, various strategies will be undertaken to address the common methodological issues related to case studies. Strategies to improve validity include triangulation of multiple data sources, member checking, data analysis in pairs, disclosure of researcher bias or reflexivity, and external validation [57].

### Conclusions

The findings of this study will extend the current understanding of PFE in digital health and will contribute broadly to health informatics, participatory medicine, patient experience, design, and implementation-science literature. The case study approach will compare the current understanding of PFE processes with the lived experience of implementing PFE, thereby exploring and uncovering the tacit nuances of initiating and integrating PFE into practice. The exploratory case study approach also offers the opportunity to further develop the conceptual and theoretical understanding of PFE in digital health [38,58], an area that has not been systematically documented or understood [59-61].

With a better understanding of how PFE occurs within digital health projects, a resource guide can be developed to support the successful uptake of these strategies by other organizations. If PFE becomes commonplace in all stages of the life cycle of digital health technologies, there may be improved uptake, satisfaction, and engagement with these technologies by patients and their family members.

## Appendix

Multimedia Appendix 1 Protocols.

## Abbreviations

CHIR: Canadian Institutes of Health Research
PFE: patient and family engagement
IT: information technology
