# Supplementary material for: Patient and Family Engagement Approaches for Digital Health Initiatives: Protocol for a Case Study
Source: JMIR Res Protoc. 2021 Jul 21;10(7):e24274. doi: 10.2196/24274 (PMC8339977; doi:10.2196/24274)
Supplement: Multimedia Appendix 1 [file resprot_v10i7e24274_app1.docx]

**Materials for Data Collection**

1. **Participation-Observation Protocol**

As a participant-observer in digital health initiatives, field notes will be taken to document the conversations and the processes involved in planning and implementing PFE activity. Areas of observation include but are not limited to the domains outlined int Table 1.

Table 1. Areas of Observation

| Domain | Description/Aims |
| --- | --- |
| PFE processes | Noted related to PFE process should be taken to understand or identify:   - Steps required in planning, implementing, and evaluating PFE - Common question and responses from the perspective of the project team and PFE coordinators - Tips from the PFE Coordinators on effective engagement - Pre-requisites, documentation, or information required to effectively engage patients - Decision points or milestones in the PFE process |
| Implementation Factors | Notes or reflections on the experience of planning, implementing, and evaluating PFE should be taken to understand or identify:   - Enablers - Barriers - Challenges/Pain points - Breakthrough moments |
| Artefacts and Documentation | *Artefacts* - technologies, templates, patient facing material, process maps, project documents, PowerPoint slides decks  *Documentation* - agendas, meeting minutes, report of events, proposals, progress reports, evaluation reports, published articles, and news appearing in institutional announcements and mass media  Notes describing the *artefact/documents* should be taken whenever one is introduced to facilitate the planning, implementation, and evaluation of PFE. Notes should include:   - Date introduced - Context where artefact was introduced - The purpose of the artefact - Team discussion about the artefact - Revisions made to the artefacts. |

1. **Interviews with digital health teams**

Script

Thank you for participating in this project.

We are developing a field guide to help CAMH staff plan and implement patient engagement strategies in the design of their digital health projects.

This project will use a case study approach to document the processes involved in planning and implementing PFE in digital health initiatives at CAMH and understand how we can build capacity at CAMH to support PFE in digital health initiatives.

We will also be recording this conversation for reference to supplement our notes. The recordings will not be transcribed only be accessed by myself. Do we have your permission?

- 1. How do your rate your PFE experience in your project?
     1. What did you like about it?
     2. What did you dislike about it?
  2. Do you think patients were effectively engaged for this project? Why?
     1. How do you think we can increase the effectiveness of the engagement?
  3. What was the value of PFE in your project?
  4. Would you include PFE in future projects or other aspects of this project?
     1. If so, in what capacity?
     2. If no, why not?
  5. What worked smoothly in your experience?
  6. What were some areas of difficulties?
     1. What was done overcome the difficulties?
  7. What is one thing you would do differently?
  8. What do you know now that you wish you knew before you started?
  9. What are some questions about PFE that you still have?
  10. What areas if PFE are interesting to you?
  11. What were some enablers for PFE in your project?
  12. What were some barriers for PFE in your project?
